# Supplementary figures and images for: An engineered biosensor enables dynamic aspartate measurements in living cells
Source: eLife. 2024 Feb 23;12:RP90024. doi: 10.7554/eLife.90024 (PMC10942590; doi:10.7554/eLife.90024)

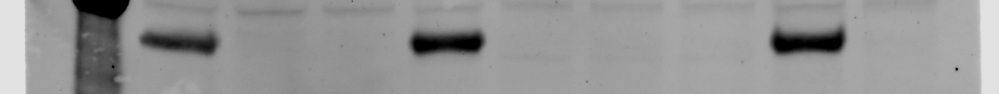

Supplement: Figure 2—source data 2. [file elife-90024-fig2-data2.zip › Fig2_SourceData2_FocusedScans/Fig2D_Blot2_GOT2scan.png]

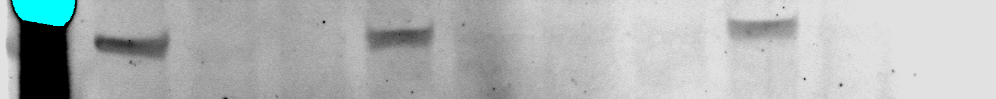

Supplement: Figure 2—source data 2. [file elife-90024-fig2-data2.zip › Fig2_SourceData2_FocusedScans/Fig2D_Blot1_GOT1scan.png]

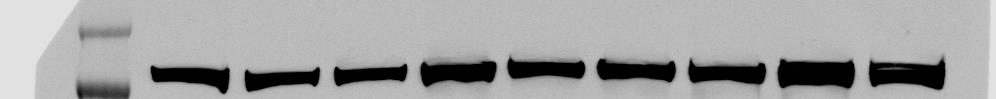

Supplement: Figure 2—source data 2. [file elife-90024-fig2-data2.zip › Fig2_SourceData2_FocusedScans/Fig2D_Blot2_Vinculinscan.png]
