# Supplementary material for: An engineered biosensor enables dynamic aspartate measurements in living cells
Source: eLife. 2024 Feb 23;12:RP90024. doi: 10.7554/eLife.90024 (PMC10942590; doi:10.7554/eLife.90024)
Supplement: Figure 2—source data 3. [file elife-90024-fig2-data3.pdf]

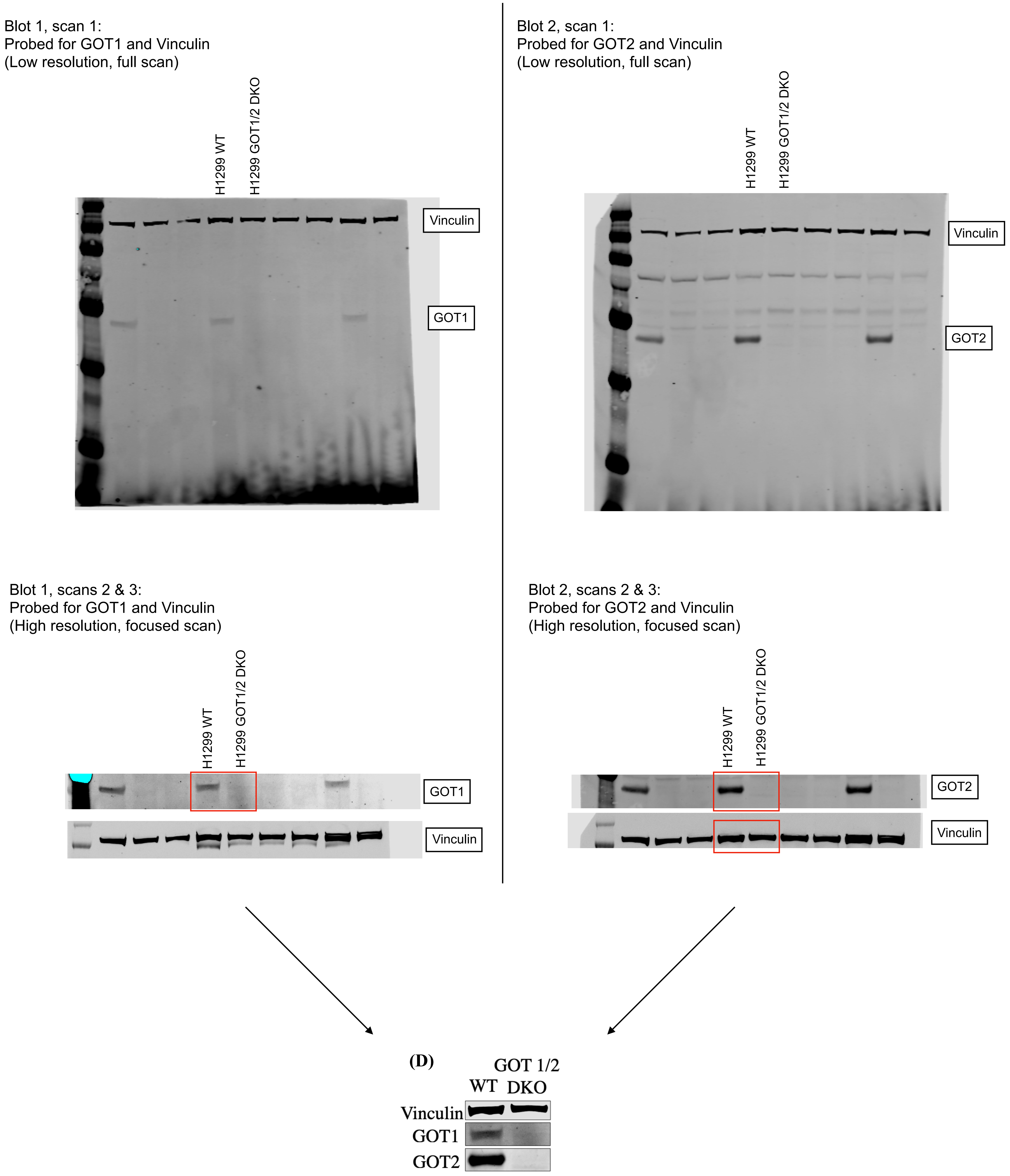
Blot 1, scan 1:
Probed for GOT1 and Vinculin
(Low resolution, full scan)
H1299 WT
H1299 GOT1/2 DKO
Vinculin
GOT1
Blot 2, scan 1:
Probed for GOT2 and Vinculin
(Low resolution, full scan)
H1299 WT
H1299 GOT1/2 DKO
Vinculin
GOT2
Blot 1, scans 2 & 3:
Probed for GOT1 and Vinculin
(High resolution, focused scan)
H1299 WT
H1299 GOT1/2 DKO
GOT1
Vinculin
Blot 2, scans 2 & 3:
Probed for GOT2 and Vinculin
(High resolution, focused scan)
H1299 WT
H1299 GOT1/2 DKO
GOT2
Vinculin
(D)
GOT 1/2
WT
DKO
Vinculin
GOT1
GOT2

Figure 2
